# Brief behaviour change counselling in the South African context

**DOI:** 10.4102/safp.v67i1.6122

**Published:** 2025-12-19

**Authors:** Zelra Malan, Robert Mash

**Affiliations:** 1 Department of Family Medicine, Faculty of Medicine, University of Namibia, Windhoek, Namibia; 2 Department of Family and Emergency Medicine, School of Medicine, Stellenbosch University, Stellenbosch, South Africa

Dear Editor,

A recently published continuing professional development article promoted the ‘5Rs approach’ to help people who are not willing to engage in behavioural change.^1^ While the 5Rs themselves appear to be reasonable recommendations, the positioning of this approach in the field of behavioural change needs correction.

The article states that motivational interviewing (MI) ‘posits that people go through six stages of change’ and that the main task of the clinician using MI is to ‘identify which stage the patient is in and motivate them to shift towards a desired change in behaviour’. In our view, this is not an accurate description of MI.

Professors William Miller and Stephen Rollnick, the founders of MI, published ‘ten things that motivational interviewing is not’ in 2009 and made it clear that MI is not based on the transtheoretical model of stages of change.^2^ The initial publications on the stages of change and MI happened more or less at the same time, but they were unrelated and MI is not based on the stages of change. They certainly speak to each other, but MI was developed out of clinical practice and not from this theory. The founders describe them ‘as kissing cousins that never married’.^2^ Motivational interviewing does not require someone to be assigned to a stage of change, and the clinical method is about enhancing personal motivation to change.^3^

The stated limitations of MI are also unsupported by evidence or argumentation and in our view are not correct. Firstly, ‘ignoring the social context’. One of the key pillars of MI is an empathic and evocative approach that seeks to understand the person’s perspective on change.^3^ This inevitably includes understanding the person’s social context, such as family, work and living conditions as these are typically part of the motivational landscape. Secondly, the article states that there is no ‘time limit on how long a patient can be in a stage’. Given the background to MI and the stages of change in the previous paragraph this limitation is not supported. The final limitation is stated as an assumption that ‘once motivated, patients can always make informed decisions’. The MI approach includes the need to inform the patient, ideally through an exchange of information, as well as respect for the person’s choice and control.^3^ It is not entirely clear what the authors regard as a limitation here.

The article also implies that the 5As approach to behaviour change can only ‘support patients who are already willing to change’. In South Africa, the 5As approach has been adapted to the guiding style, derived from MI.^4^ This adapted approach has the following steps: ask, alert, assess, assist and arrange. The approach has a strong evidence base within established and effective training, and assessment methods.^5,6,7,8,9^ This work performed in South Africa is not referenced or discussed in the article. In fact, the reference given for the 5As does not mention them at all and is an article on MI for the competent novice.^10^ The locally adapted 5As approach is designed for individuals with varying readiness to change, offering tailored strategies to support them based on their level of readiness. Positioning the 5As as an approach that is only for people who are ready to change is not correct.

Having said all this, the actual 5R strategies (relevance, risks, rewards, roadblocks, repetition) are reasonable and are already part of the locally adapted 5As approach. The 5As include a step on alerting the person to the risks of continuing the behaviour and the rewards of changing and includes the need to help the person reflect on the relevance of this information. The approach includes the need to anticipate and plan for roadblocks or challenges in making a change. The approach also includes follow up, ongoing support and repeated interactions.

In conclusion, the 5R strategies are already part of the 5As approach in the South African context and should not be regarded as an alternative or competing method. Integration of the 5Rs and the 5As has also been suggested elsewhere.^11^ Please study the references below for further information.
